# Metabolic syndrome confers a higher risk of oral cancer: a meta-analysis

**DOI:** 10.3389/fonc.2026.1919070

**Published:** 2026-08-21

**Authors:** Lijun Liao, Juan Nie, Ru Jia

**Affiliations:** 1 Department of Orthodontics, Sichuan Hospital of Stomatology, Chengdu, China; 2 Department of Pediatric Dentistry, Sichuan Hospital of Stomatology, Chengdu, China

**Keywords:** incidence, metabolic syndrome, oral cancer, oral squamous cell carcinoma, risk factor

## Abstract

**Background:**

Metabolic syndrome (MetS) has been implicated in the development of several malignancies, but its association with oral cancer (OC) remains uncertain. This meta-analysis aimed to evaluate the association between MetS and the risk of incident OC.

**Methods:**

PubMed, Embase, and Web of Science were systematically searched from inception to May 11, 2026. Longitudinal cohort studies comparing the incidence of OC between adults with and without MetS were included. Hazard ratios (HRs) and 95% confidence intervals (CIs) were pooled using random-effects models accounting for potential heterogeneity.

**Results:**

Four cohort studies comprising 6,370,396 participants and 4,539 incident OC cases were included. One study reported sex-specific estimates, resulting in five datasets for the primary meta-analysis. Overall, MetS was associated with a significantly increased risk of OC (HR: 1.12, 95% CI: 1.04–1.20; *p* = 0.001), with low heterogeneity (I² = 8%). Leave-one-out sensitivity analyses yielded consistent results (pooled HRs: 1.08–1.16). Subgroup analysis showed no significant difference between men and women (HR: 1.14 vs. 1.02; *p* for subgroup difference = 0.35). Exploratory analyses indicated that hypertriglyceridemia (HR: 1.07, 95% CI: 1.00–1.14; *p* = 0.04) and hyperglycemia (HR: 1.22, 95% CI: 1.10–1.36; *p* < 0.001) were significantly associated with increased OC risk, whereas central obesity, low high-density lipoprotein cholesterol, and hypertension were not.

**Conclusions:**

Current longitudinal evidence suggests that MetS is associated with a modest but significant increase in the risk of incident OC. Findings for individual MetS components are exploratory and hypothesis-generating.

**Systematic review registration:**

https://www.crd.york.ac.uk/PROSPERO/, identifier CRD420261432959.

## Introduction

Oral cancer (OC), of which oral squamous cell carcinoma accounts for more than 90% of cases, is one of the most common malignancies of the head and neck and remains a major global health challenge because of its considerable morbidity and mortality (1, 2). Despite advances in surgery, radiotherapy, chemotherapy, targeted therapy, and immunotherapy, the overall prognosis of patients with advanced OC remains unsatisfactory, with five-year survival rates remaining below 50% in many populations (3, 4). In addition to its adverse impact on survival, OC frequently results in substantial impairments in speech, swallowing, mastication, facial appearance, and quality of life, imposing a considerable burden on patients and healthcare systems (5). Well-established risk factors for OC include tobacco smoking, excessive alcohol consumption, betel quid chewing, poor oral hygiene, chronic inflammation, and human papillomavirus (HPV) infection, although these factors do not fully explain disease occurrence (6, 7). Increasing evidence suggests that systemic metabolic disturbances may contribute to oral carcinogenesis beyond the established behavioral risk factors (8, 9). Experimental and epidemiological studies indicate that chronic inflammation, insulin resistance, oxidative stress, adipokine dysregulation, and metabolic reprogramming associated with MetS may promote epithelial proliferation, inhibit apoptosis, facilitate genomic instability, and create a tumor-promoting microenvironment (10, 11). Therefore, identifying potentially modifiable metabolic risk factors may improve risk stratification and facilitate the development of preventive strategies for this highly burdensome malignancy.

Metabolic syndrome (MetS) is a cluster of interrelated metabolic abnormalities characterized by central obesity, hyperglycemia, hypertension, high triglyceride (TG), and reduced high-density lipoprotein cholesterol (HDL-C), and its prevalence has increased substantially worldwide in parallel with the obesity epidemic (12–14). Beyond its well-established association with cardiovascular disease and type 2 diabetes (15), MetS has been increasingly recognized as a potential contributor to cancer development through mechanisms involving chronic low-grade inflammation, insulin resistance, oxidative stress, dysregulated insulin-like growth factor signaling, adipokine imbalance, and immune dysfunction (16–18). However, the relationship between MetS and cancer appears to vary according to tumor site, with stronger associations reported for certain gastrointestinal, hepatobiliary, and gynecological malignancies (19, 20) than for others (21, 22). Several longitudinal cohort studies have evaluated the association between MetS and the risk of OC (23–26), but the results remain inconsistent, with some reporting a significantly increased risk and others observing no independent association. Moreover, the contributions of individual MetS components to OC risk remain unclear. To date, no meta-analysis has comprehensively synthesized the available longitudinal evidence. Therefore, we conducted this systematic review and meta-analysis to quantitatively evaluate the association between MetS and the risk of incident OC, explore potential sex-specific differences, and examine the associations between individual MetS components and OC risk.

## Methods

This meta-analysis was conducted in accordance with recognized standards for systematic reviews and meta-analyses. The methodology adhered to the recommendations of the PRISMA 2020 guideline (27) and the Cochrane Handbook for Systematic Reviews of Interventions (28), covering all stages from protocol development and literature screening to data extraction, quantitative synthesis, and interpretation of findings. The review protocol was prospectively registered in PROSPERO (CRD420261432959).

### Database search

A comprehensive search of the literature was performed in PubMed, Embase, and Web of Science to identify all relevant studies, using a combination of the following search terms (1) “metabolic syndrome” OR “insulin resistance syndrome” OR “syndrome X”; and (2) “oral squamous cell carcinoma” OR “oral cancer” OR “oral cavity cancer” OR “mouth neoplasm”. Eligibility was restricted to peer-reviewed full-text studies involving human participants and published in English. To ensure completeness, the reference lists of relevant reviews and included articles were also screened manually for additional records. The search was restricted to peer-reviewed, English-language publications, which comprehensively cover the majority of high-quality biomedical literature. Grey literature was not included because it often provides insufficient methodological and outcome data for quantitative synthesis and has not undergone formal peer review. Database searches covered the period from database inception to May 11, 2026. The complete search strategies used for each database are provided in **Supplementary File 1**.

### Study inclusion and exclusion criteria

The eligibility criteria were established according to the Population, Exposure, Comparator, Outcome, and Study design (PICOS) framework.

Population (P): Adult participants (aged ≥ 18 years) without a prior diagnosis of OC at baseline were eligible. Studies conducted in the general population or specific adult populations were included.

Exposure (I/E): The exposure of interest was MetS, defined according to established diagnostic criteria, including the National Cholesterol Education Program Adult Treatment Panel III (NCEP-ATP III), the International Diabetes Federation (IDF), the American Heart Association/National Heart, Lung, and Blood Institute (AHA/NHLBI), the Joint Interim Statement (JIS), or other established diagnostic criteria for MetS.

Comparator (C): Participants without MetS, as defined by the corresponding diagnostic criteria used in each study, served as the comparator group.

Outcomes (O): The primary outcome was incident OC, identified by pathological confirmation, cancer registry records, or validated diagnostic codes, compared between adults with and without MetS at baseline.

Study Design (S): Prospective or retrospective longitudinal cohort studies reporting associations between MetS and subsequent risk of OC were eligible.

Studies were excluded if they (1) enrolled children or adolescents (< 18 years); (2) evaluated composite metabolic risk scores, or metabolically healthy/unhealthy phenotypes without a formal diagnosis of MetS; (3) reported outcomes other than incident OC (e.g., oral potentially malignant disorders [OPMDs], head and neck cancer without separate data of OC incidence, or cancer mortality); (4) were cross-sectional studies, case–control studies, case reports, case series, reviews, editorials, animal studies, or *in vitro* studies; (5) did not report effect estimates or provide sufficient data for their calculation; or (6) included duplicate or overlapping populations. In cases of overlapping populations, only the most comprehensive study, generally the one with the longest follow-up duration and most complete adjusted analysis, was included.

### Study quality assessment

All stages of the review process were conducted independently by two investigators, including study identification, eligibility assessment, data extraction, and quality appraisal. Discrepancies were resolved through discussion, with adjudication by a third reviewer when consensus could not be achieved. Study quality was assessed using the Newcastle–Ottawa Scale (NOS) (29), which evaluates the risk of bias in observational studies across participant selection, group comparability, and outcome ascertainment. Scores range from 0 to 9, with a score of 7 or higher indicating high methodological quality.

### Data collection

Two reviewers independently extracted data from eligible studies using a standardized data extraction form developed *a priori*. The extracted information included study characteristics (first author, publication year, country, and study design), participant characteristics (source of population, sample size, mean age, and sex distribution), diagnostic criteria for MetS and number of adults with MetS at baseline, mean follow-up durations, methods for the diagnosis of OC, number of patients who developed OC during follow-up, and variables included in multivariable-adjusted analyses evaluating the association between MetS and the incidence of OC.

### Statistical analyses

The association between MetS and the risk of OC was quantified by pooling hazard ratios (HRs) and corresponding 95% confidence intervals [CIs], comparing participants with and without MetS at baseline (28). When multiple adjusted models were reported, the estimates derived from the model with the most comprehensive adjustment were selected for analysis. For studies reporting only sex-specific estimates without an overall effect estimate, each sex-specific cohort was treated as an independent, non-overlapping population because participants contributed to only one subgroup. If required, effect sizes and standard errors were calculated from the available 95% CIs or *p* values. Prior to data synthesis, all effect estimates were transformed to the natural logarithmic scale to improve distribution normality and stabilize variances (28). Between-study inconsistency was examined using Cochran’s Q statistic and quantified with the I² metric (30). Heterogeneity was interpreted as low, moderate, or substantial when I² values were < 25%, 25–75%, and > 75%, respectively. Given the anticipated clinical and methodological diversity among studies, pooled estimates were generated using an inverse-variance random-effects model based on the DerSimonian–Laird method (28). The robustness of the findings was investigated through leave-one-out sensitivity analyses, whereby the meta-analysis was repeated after sequential removal of each individual study (31). In addition, prespecified subgroup analyses by sex were conducted. Moreover, an exploratory meta-analysis was also performed to evaluate the associations of components of MetS and the risk of OC, including central obesity, high TG, low HDL-C, hyperglycemia, and hypertension. Potential small-study effects and publication bias were explored by visual assessment of funnel plots and formally tested using Egger’s linear regression method (32). Statistical significance was determined using a two-tailed *p* value < 0.05. All quantitative analyses were carried out using RevMan version 5.3 (Cochrane Collaboration, Oxford, UK) and Stata version 17.0 (StataCorp, College Station, TX, USA).

### Certainty of evidence assessment

The certainty of evidence for the primary outcome was evaluated using the Grading of Recommendations Assessment, Development and Evaluation (GRADE) approach (33). The certainty of evidence was assessed across the domains of risk of bias, inconsistency, indirectness, imprecision, and publication bias and categorized as high, moderate, low, or very low (33). Because all included studies were observational cohort studies, the certainty of evidence was initially rated as low and subsequently evaluated across the remaining GRADE domains.

## Results

### Database search results

**Figure 1:**
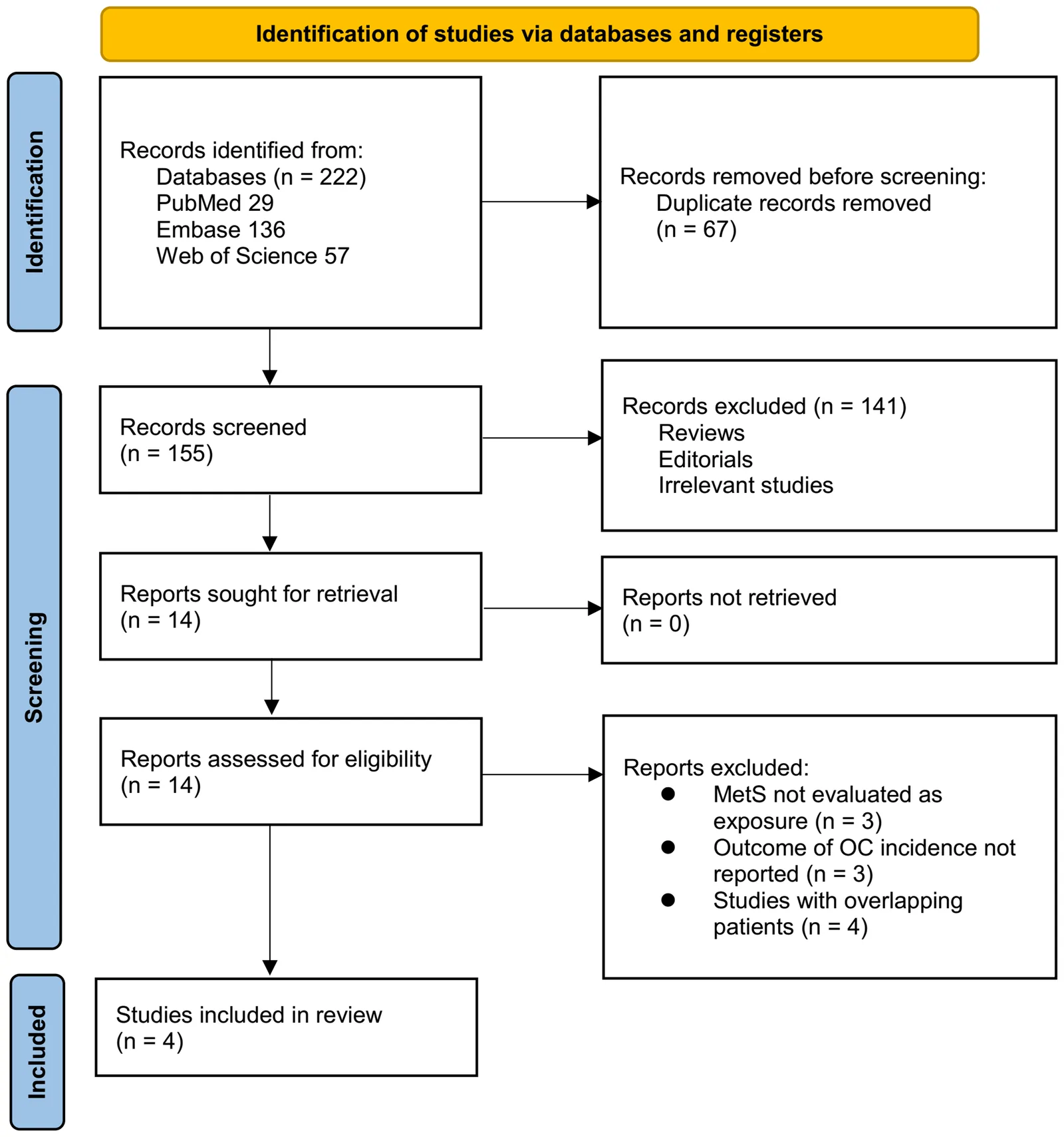
Flow diagram of the study selection process.

**Figure 1** summarizes the study identification and selection process. The database search yielded 222 potentially relevant records, of which 67 were duplicates and subsequently removed. Screening of the remaining citations resulted in the exclusion of 141 records at the title and abstract stage. Fourteen articles were retrieved for detailed full-text assessment, and 10 were excluded after eligibility evaluation. Ultimately, four studies satisfied the inclusion criteria and were included in the meta-analysis (23–26).

### Summary of study characteristics

The main characteristics of the included studies are summarized in **Table 1**. Four cohort studies published between 2013 and 2025 were included, comprising 6,370,396 participants. Of these, two studies adopted a prospective cohort design (25, 26), whereas the remaining two were retrospective cohort studies (23, 24). The studies were conducted in three countries, including the Netherlands, Korea, and the United Kingdom, indicating moderate geographic diversity. The mean age of participants ranged from 53.5 to 59.7 years, while the proportion of men ranged from 48.6% to 74.3%. Two studies enrolled participants from the general adult population (24, 25), one included patients with vascular disease (26), and one enrolled hepatitis B virus carriers (23). MetS was defined using the NCEP-ATP III (25, 26), the JIS (23), or the IDF (24) criteria. Overall, 1,848,191 adults (29.0%) had MetS at baseline. Mean follow-up durations ranged from 5.5 to 11.7 years, during which 4,539 incident OC cases were identified. OC was consistently ascertained using International Classification of Diseases, Tenth Revision (ICD-10) diagnostic codes (**Supplementary Table 1**). All included studies performed multivariable-adjusted analyses, with adjustment for important confounding factors, commonly including age, sex, smoking status, alcohol consumption, body mass index, and other demographic or lifestyle characteristics to a varying degree.

**Table 1:** Characteristics of the included studies.

Study	Country	Study design	Population source	No. of participants	Mean age (years)	Men (%)	Diagnostic criteria of MetS	No. of participants with MetS	Mean follow-up (years)	No. of patients with OC	Methods for validation of OC	Variables adjusted
van Kruijsdijk 2013 (26)	The Netherlands	PC	Patients with vascular disease	6,172	59.7	74.3	NCEP-ATP III	3,301	5.5	18	ICD-10 codes	Age, sex, BMI, smoking status, and alcohol drinking
Choe 2021 (23)	Korea	RC	HBV Carriers	1,504,880	53.5	56.6	JIS	582,449	7	1,459	ICD-10 codes	Age, sex, BMI, smoking status, alcohol drinking, and physical activity
Choi 2023 (24)	Korea	RC	General adult population aged over 40 years	4,569,787	54	54.1	IDF	1,195,476	10	2,718	ICD-10 codes	Age, sex, smoking, and alcohol drinking
Lin 2025 (25)	UK	PC	General adult population	289,557	56.1	48.6	NCEP-ATP III	66,965	11.7	344	ICD-10 codes	Age, sex, ethnicity, family cancer history, Townsend deprivation index, and education

None of the included studies adjusted for HPV infection or betel quid chewing.

PC, prospective cohort; RC, retrospective cohort; MetS, metabolic syndrome; OC, oral cancer; HBV, hepatitis B virus; BMI, body mass index; ICD-10, International Classification of Diseases, Tenth Revision; NCEP-ATP III, National Cholesterol Education Program Adult Treatment Panel III; JIS, Joint Interim Statement; IDF, International Diabetes Federation.

### Study quality evaluation

The methodological quality of the included studies was assessed using the NOS, and the results are presented in **Table 2**. NOS scores ranged from 7 to 8, indicating generally high methodological quality. All studies appropriately selected comparison cohorts, used validated criteria to ascertain MetS, confirmed the absence of OC at baseline, adjusted for important confounding factors, and provided adequate follow-up. No study received a score for independent outcome assessment because OC was identified using ICD-10 diagnostic codes from national administrative or registry databases. In addition, two retrospective studies (23, 24) lost one point because the exposed cohorts were not considered fully representative of the general population. Overall, the methodological quality of the included studies supports the reliability of the pooled estimates.

**Table 2:** Study quality evaluation via the Newcastle-Ottawa Scale.

Study	Representativeness of the exposed cohort	Selection of the non-exposed cohort	Ascertainment of exposure	Outcome not present at baseline	Control for age and sex	Control for other confounding factors	Assessment of outcome	Enough long follow-up duration	Adequacy of follow-up of cohorts	Total
van Kruijsdijk 2013 (26)	1	1	1	1	1	1	0	1	1	8
Choe 2021 (23)	0	1	1	1	1	1	0	1	1	7
Choi 2023 (24)	0	1	1	1	1	1	0	1	1	7
Lin 2025 (25)	1	1	1	1	1	1	0	1	1	8

### Association between MetS and the incidence of OC

**Figure 2:**
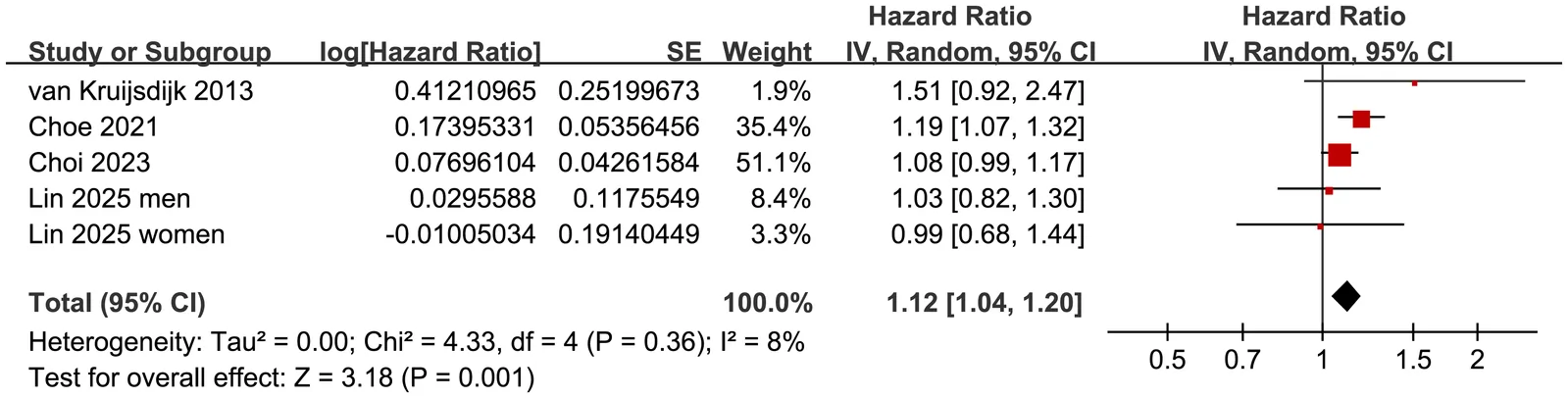
Forest plots showing the meta-analysis of the association between MetS and the risk of OC.

**Figure 3:**
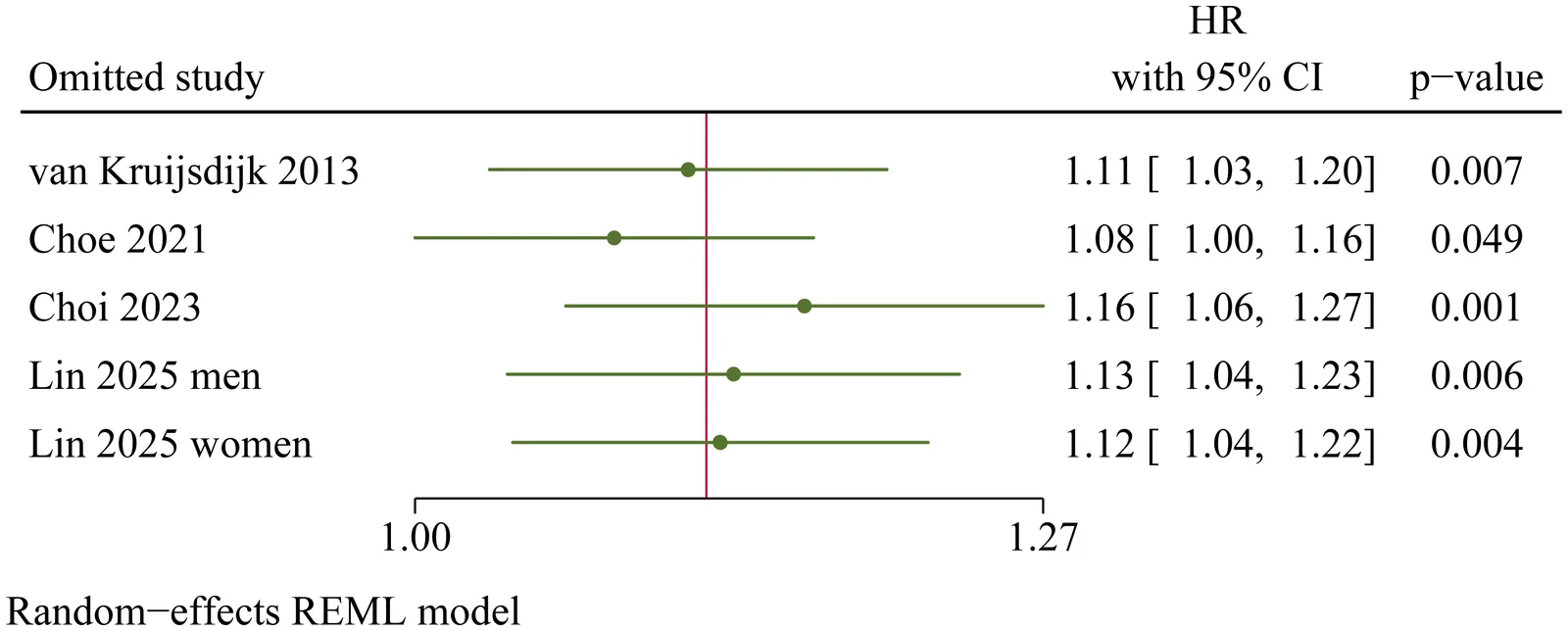
Leave-one-out sensitivity analysis of the association between MetS and the risk of OC.

**Figure 4:**
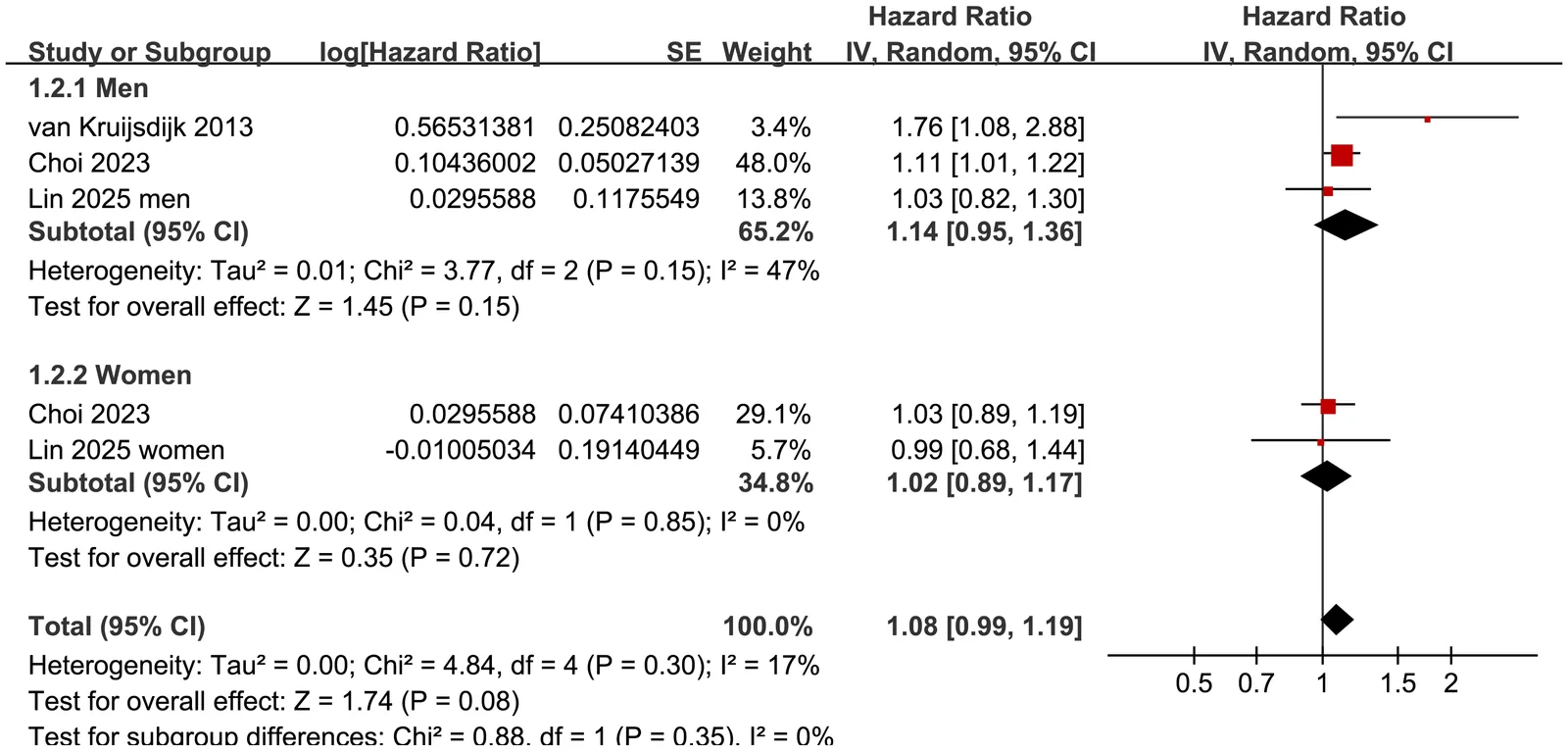
Forest plots showing the subgroup analysis by sex.

**Figure 5:**
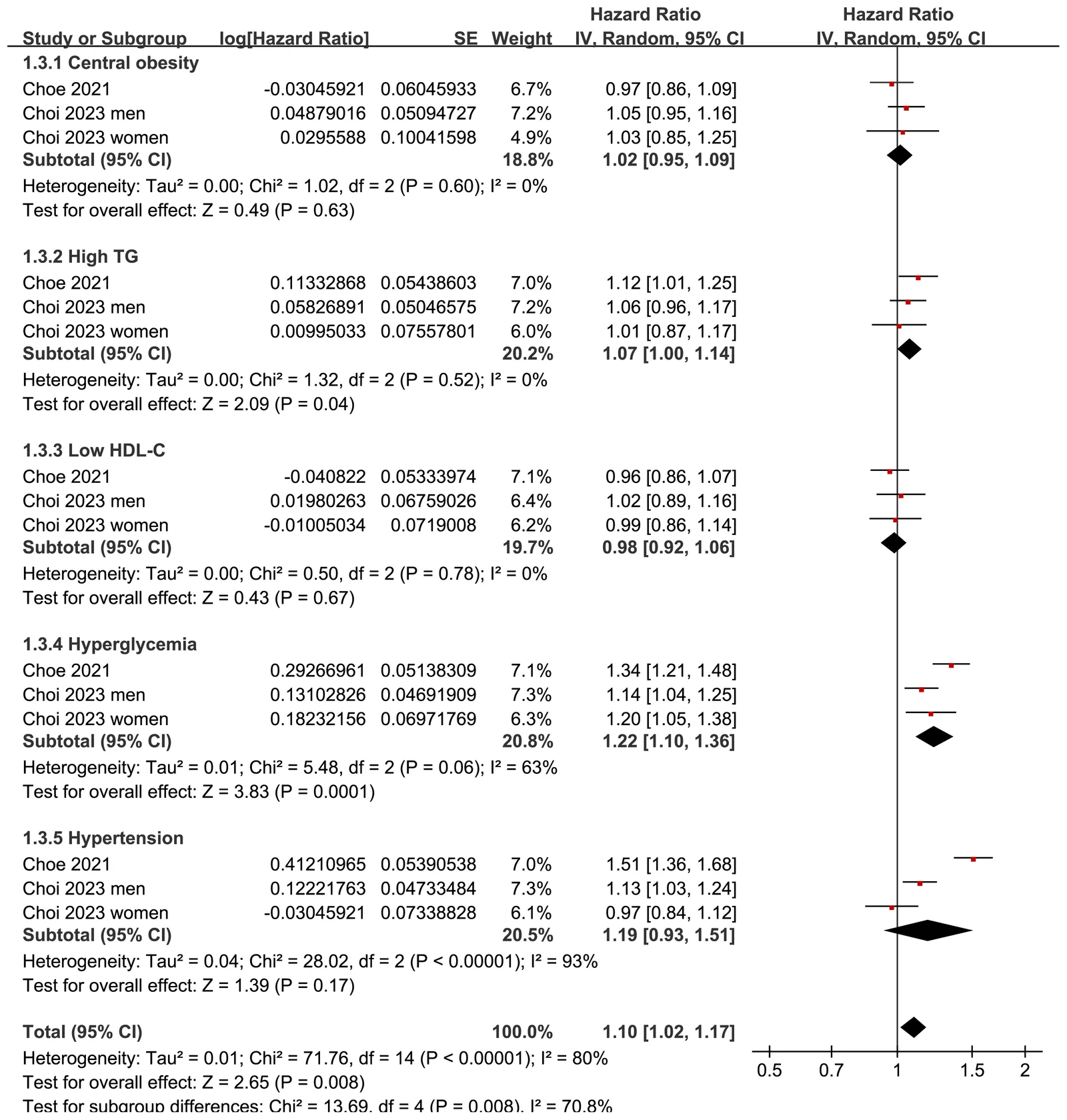
Forest plots showing the exploratory meta-analyses of the associations between individual MetS components and the risk of OC.

One study reported sex-specific rather than overall estimates of the association between MetS and the risk of OC (25). Therefore, the male and female datasets were treated as independent cohorts and included separately in the meta-analysis. The pooled analysis of the four studies (23–26) showed that MetS was associated with an increased risk of OC among adults (HR: 1.12, 95% CI: 1.04 to 1.20, *p* = 0.001; **Figure 2**) without significant heterogeneity (*p* for Cochrane Q test = 0.36; I^2^ = 8%). Leave-one-out sensitivity analyses yielded consistent findings, with pooled HRs ranging from 1.08 to 1.16 and all corresponding *p* values < 0.05 (**Figure 3**), which indicated that no single study substantially influenced the overall result and supported the robustness of the pooled estimate. In an additional sensitivity analysis restricted to general-population cohorts, two studies (24, 25) contributing three independent datasets were included. The pooled association was attenuated and did not reach statistical significance (HR: 1.07, 95% CI: 0.99 to 1.16; *p* = 0.08), with no evidence of heterogeneity (I² = 0%). Further subgroup analysis showed no statistically significant difference in the association between MetS and the risk of OC between men and women (HR: 1.14 vs. 1.02; *p* for subgroup difference = 0.35; **Figure 4**). However, these findings should be interpreted cautiously because only two to three datasets were available for each subgroup, limiting the statistical power to detect potential sex-specific differences. Finally, exploratory meta-analyses, each based on three datasets, showed that among the individual components of MetS, hypertriglyceridemia (HR: 1.07, 95% CI: 1.00 to 1.14, *p* = 0.04) and hyperglycemia (HR: 1.22, 95% CI: 1.10 to 1.36, *p* < 0.001) were significantly associated with an increased risk of OC. In contrast, no significant associations were observed for central obesity, low HDL-C, or hypertension (all *p* > 0.05; **Figure 5**).

### Publication bias

Publication bias could not be reliably assessed because only four studies (five datasets) were available, which is below the recommended number for meaningful assessment of publication bias. Accordingly, no funnel plot was generated, and publication bias was considered not assessable.

### GRADE assessment

The GRADE assessment is presented in **Table 3**. The certainty of evidence for the association between MetS and incident OC was rated as low. The evidence was not further downgraded because the included studies were generally of high methodological quality, between-study heterogeneity was low, the evidence directly addressed the review question, and the pooled estimate was considered sufficiently precise. Publication bias could not be reliably assessed because only four studies (five datasets) were available.

**Table 3:** GRADE assessment of the certainty of evidence for the association between MetS and the risk of OC.

Outcome	No. of studies (cohorts)	Participants	Study design	Risk of bias	Inconsistency	Indirectness	Imprecision	Publication bias	Effect estimate/Certainty
Incident OC	4 studies (5 datasets)	6,370,396	Observational cohort studies	Not serious^1^	Not serious²	Not serious^3^	Not serious^4^	Not assessable^5^	HR 1.12 (95% CI 1.04–1.20) Low ⊕⊕◯◯

Explanations.

¹Risk of bias.

The evidence was derived exclusively from observational cohort studies, which begin at low certainty under the GRADE framework. All included studies were of high methodological quality according to the Newcastle–Ottawa Scale, and most reported multivariable-adjusted hazard ratios. Therefore, no further downgrading for risk of bias was applied.

²Inconsistency.

Between-study heterogeneity was low, and the direction and magnitude of the association remained generally consistent across subgroup and sensitivity analyses.

³Indirectness.

The populations, exposure, comparator, and outcome directly addressed the review question. Although two studies enrolled selected clinical populations, they evaluated the same predefined exposure–outcome relationship and were therefore not considered to introduce serious indirectness.

^4^Imprecision.

The pooled estimate was based on 6,336,794 participants and 4,539 incident oral cancer cases, with a relatively narrow confidence interval excluding the null value.

^5^Publication bias.

Publication bias could not be reliably assessed because only four studies (five datasets) were available, which is below the recommended number for meaningful interpretation of funnel plot asymmetry or statistical tests for small-study effects. Therefore, this domain was judged as not assessable rather than downgraded.

## Discussion

In this meta-analysis of longitudinal cohort studies involving more than 6.3 million participants, we found that MetS was associated with an increased risk of incident OC. The association remained robust in leave-one-out sensitivity analyses, indicating that the overall findings were not driven by any single study. Moreover, exploratory analyses suggested that hyperglycemia and high TG, but not the other MetS components, were associated with an increased risk of OC. Although subgroup analysis did not reveal a significant difference between men and women, the limited number of available datasets warrants cautious interpretation. Collectively, these findings support MetS as a potential metabolic risk factor for OC and provide biological plausibility for the hypothesis that chronic metabolic dysregulation—including persistent inflammation, insulin resistance, oxidative stress, adipokine imbalance, and altered glucose and lipid metabolism—may collectively create a microenvironment conducive to oral carcinogenesis.

Several biological mechanisms may explain the observed association between MetS and OC. MetS is characterized by chronic low-grade systemic inflammation, insulin resistance, visceral adiposity, oxidative stress, and dysregulated lipid metabolism, all of which may promote tumor initiation and progression (34, 35). Persistent inflammation leads to increased circulating concentrations of pro-inflammatory cytokines, including tumor necrosis factor-α, interleukin-6, and C-reactive protein, which activate signaling pathways involved in cellular proliferation, angiogenesis, epithelial–mesenchymal transition, and inhibition of apoptosis (36, 37). Insulin resistance and compensatory hyperinsulinemia further stimulate insulin and insulin-like growth factor-1 signaling, thereby enhancing cellular proliferation and suppressing apoptosis through activation of the phosphatidylinositol 3-kinase/Akt and mitogen-activated protein kinase pathways (38, 39). In addition, obesity-related adipokine imbalance, characterized by increased leptin and reduced adiponectin concentrations, may create a pro-tumorigenic microenvironment by promoting inflammation, angiogenesis, and immune dysregulation (40). Oxidative stress associated with MetS may further induce DNA damage, genomic instability, and epigenetic alterations that facilitate malignant transformation (41). These mechanisms are biologically plausible and are consistent with previous evidence linking MetS to the development of several malignancies, although the magnitude of the association appears to vary according to cancer site (21).

Our exploratory component analyses provide possible insight into the pathways underlying the observed association, but they should not be interpreted as establishing causality. Hyperglycemia may plausibly promote carcinogenesis through oxidative stress, accumulation of advanced glycation end products, chronic inflammatory signaling, and activation of insulin and insulin-like growth factor pathways (42, 43), whereas hypertriglyceridemia may contribute through lipid peroxidation, altered membrane composition, inflammation, and the provision of metabolic substrates for proliferating tumor cells (44). However, the significant associations observed for hyperglycemia and high TG were based on only three datasets, and the individual components of MetS are biologically and statistically interrelated. Therefore, these findings cannot determine whether glucose and triglyceride abnormalities directly mediate oral carcinogenesis or instead reflect the broader metabolic dysfunction represented by MetS. Similarly, the absence of significant associations for central obesity, hypertension, and low HDL-C should not be interpreted as evidence that these factors have no biological relevance, because limited statistical power, differences in component definitions, and adjustment for correlated metabolic variables may have attenuated their associations.

The association is also supported indirectly by epidemiological studies of OPMDs, which are recognized precursors of OC. These studies have reported associations of MetS, hyperglycemia, and hypertriglyceridemia with the development of premalignant oral lesions, including after adjustment for several traditional behavioral risk factors (45–47). Such findings are compatible with the possibility that metabolic dysregulation influences oral carcinogenesis from an early stage. Nevertheless, this evidence remains observational and cannot exclude residual confounding, particularly by tobacco smoking, alcohol consumption, betel quid chewing, dietary patterns, oral hygiene, socioeconomic status, and HPV infection. A continuing controversy is therefore whether MetS exerts a direct etiopathogenic effect or primarily serves as a marker of a broader adverse metabolic, inflammatory, and behavioral risk profile. Differences in MetS definitions, study populations, and covariate adjustment further complicate causal interpretation.

The subgroup analysis showed comparable associations in men and women, without significant evidence of effect modification by sex. This finding may indicate that the carcinogenic influence of MetS is not strongly sex dependent and that similar metabolic pathways operate in both sexes. However, only two to three datasets contributed to each subgroup, resulting in limited statistical power to detect modest sex-specific differences. Likewise, the consistent findings observed in leave-one-out sensitivity analyses strengthen the robustness of the primary results by demonstrating that no individual study disproportionately influenced the pooled estimate. The low between-study heterogeneity further suggests that, despite differences in study populations and MetS definitions, the direction of the association was generally consistent across cohorts. Although the primary analysis suggested a modest association between MetS and increased OC risk, this association was attenuated and became statistically non-significant when the analysis was restricted to general-population cohorts. This finding raises the possibility that differences in underlying population characteristics may have influenced the pooled estimate. Nevertheless, the restricted analysis included only two studies and therefore had limited statistical power, so the absence of statistical significance should not be interpreted as definitive evidence of no association.

This study has several important strengths. To our knowledge, this is the first meta-analysis to comprehensively evaluate the association between MetS and incident OC using exclusively longitudinal cohort studies, thereby minimizing the risk of reverse causation compared with cross-sectional or case–control designs. The literature search was comprehensive and up to date, and all included studies performed multivariable-adjusted analyses to reduce the influence of major confounding factors. Furthermore, sensitivity analyses, subgroup analyses, and exploratory analyses of individual MetS components consistently supported the primary findings, increasing confidence in the robustness of the overall conclusions.

Several limitations should also be acknowledged. First, two of the included studies were retrospective cohorts, making them susceptible to selection and information bias (48). Second, only four studies were eligible for inclusion, limiting the statistical power of subgroup analyses, precluding reliable assessment of publication bias, and preventing more detailed exploration of potential sources of heterogeneity. Although two included studies were derived from the Korean National Health Insurance Service database (23, 24), they enrolled substantially different source populations with distinct inclusion criteria, making substantial participant overlap unlikely. Nevertheless, complete exclusion of overlap cannot be verified, although leave-one-out sensitivity analyses demonstrated that omission of either study did not materially influence the pooled estimates. Moreover, second, only peer-reviewed English-language studies indexed in major international databases were included. Although these databases capture the majority of high-quality biomedical research, potentially relevant studies published in other languages or grey literature may have been missed. Third, all studies identified OC using ICD diagnostic codes obtained from administrative or registry databases rather than pathological review, which may have introduced some degree of outcome misclassification despite the generally high validity of cancer registry diagnoses (49). Fourth, differences in participant characteristics, diagnostic criteria for MetS, follow-up duration, and variables included in multivariable adjustment may have contributed to residual clinical heterogeneity. Fifth, although all included studies adjusted for several important confounding variables, residual confounding cannot be excluded because adjustment for certain established oral cancer risk factors, including dietary habits, oral hygiene, socioeconomic status, HPV infection (50), betel quid chewing, and other lifestyle or unmeasured factors, was incomplete or unavailable across studies. Sixth, the GRADE assessment indicated low-certainty evidence, suggesting that although the current findings support an association between MetS and incident OC, additional well-designed prospective cohort studies in diverse populations are warranted to further strengthen the certainty of the evidence. Finally, as with all observational studies, the present meta-analysis demonstrates an association rather than a causal relationship between MetS and OC.

The present findings may have several clinical implications. Given the high and increasing global prevalence of MetS, early identification and appropriate management of metabolic abnormalities may represent an additional strategy for reducing the burden of OC, particularly among individuals with established behavioral risk factors. The observed associations with hyperglycemia and high TG further suggest that optimizing glucose and lipid metabolism could potentially contribute to oral cancer prevention, although interventional evidence remains unavailable. Future large-scale prospective studies involving diverse populations should evaluate whether the association differs according to MetS severity, individual metabolic phenotypes, lifestyle factors, and oral cancer subtypes. Mechanistic studies are also warranted to clarify the molecular pathways linking metabolic dysregulation with oral carcinogenesis and to determine whether effective treatment of MetS can reduce the incidence of OC.

## Conclusions

In conclusion, the current longitudinal evidence suggests that MetS is associated with a modest increased risk of OC. Exploratory analyses further suggested that hyperglycemia and high TG may be associated with an increased risk of OC. However, these findings should be considered hypothesis-generating because they were based on a limited number of studies evaluating highly interrelated MetS components. Given the observational nature of the available evidence, these associations should be interpreted cautiously and do not establish causality. Further high-quality prospective studies are warranted to confirm these associations and elucidate the underlying biological mechanisms.

## Data Availability

The original contributions presented in the study are included in the article/**Supplementary Material**. Further inquiries can be directed to the corresponding author.
