# Supplementary material for: Metabolic syndrome confers a higher risk of oral cancer: a meta-analysis
Source: Front Oncol. 2026 Aug 21;16:1919070. doi: 10.3389/fonc.2026.1919070 (PMC13541508; doi:10.3389/fonc.2026.1919070)
Supplement: Supplementary file 1 [file Table1.docx]

**Detailed search strategy for each database**

**PubMed**

("Metabolic Syndrome"[Mesh] OR "metabolic syndrome"[tiab] OR "metabolic syndrome X"[tiab] OR "syndrome X"[tiab] OR "insulin resistance syndrome"[tiab] OR "cardiometabolic syndrome"[tiab] OR "metabolic abnormalities"[tiab]) AND ("Oral Neoplasms"[Mesh] OR "oral cancer"[tiab] OR "oral carcinoma"[tiab] OR "oral neoplasm*"[tiab] OR "oral malignan*"[tiab] OR "oral squamous cell carcinoma"[tiab] OR OSCC[tiab] OR "mouth cancer"[tiab] OR "mouth neoplasm*"[tiab] OR "oral cavity cancer"[tiab] OR "oral cavity carcinoma"[tiab] OR "lip and oral cavity cancer"[tiab])

**Embase**

('metabolic syndrome'/exp OR 'metabolic syndrome':ti,ab OR 'metabolic syndrome x':ti,ab OR 'syndrome x':ti,ab OR 'insulin resistance syndrome':ti,ab OR 'cardiometabolic syndrome':ti,ab OR 'metabolic abnormalities':ti,ab) AND ('oral neoplasm'/exp OR 'oral cavity cancer'/exp OR 'oral squamous cell carcinoma'/exp OR 'oral cancer':ti,ab OR 'oral carcinoma':ti,ab OR 'oral neoplasm*':ti,ab OR 'oral malignan*':ti,ab OR 'oral squamous cell carcinoma':ti,ab OR OSCC:ti,ab OR 'mouth cancer':ti,ab OR 'mouth neoplasm*':ti,ab OR 'oral cavity cancer':ti,ab OR 'oral cavity carcinoma':ti,ab)

**Web of Science**

TS=(("metabolic syndrome" OR "metabolic syndrome*" OR "metabolic syndrome X" OR "syndrome X" OR "insulin resistance syndrome" OR "cardiometabolic syndrome" OR "metabolic abnormalities") AND ("oral cancer" OR "oral cancers" OR "oral carcinoma" OR "oral carcinomas" OR "oral neoplasm*" OR "oral malignan*" OR "oral squamous cell carcinoma" OR OSCC OR "mouth cancer" OR "mouth cancers" OR "oral cavity cancer" OR "oral cavity cancers" OR "oral cavity carcinoma" OR "oral cavity carcinomas" OR "lip and oral cavity cancer"))
