# Supplementary material for: Metabolic syndrome confers a higher risk of oral cancer: a meta-analysis
Source: Front Oncol. 2026 Aug 21;16:1919070. doi: 10.3389/fonc.2026.1919070 (PMC13541508; doi:10.3389/fonc.2026.1919070)
Supplement: Supplementary file 2 [file Table2.docx]

**Supplemental Table 1** ICD-10 definitions of oral cancer outcomes used in the included studies

| Study | Outcome definition |
| --- | --- |
| **van Kruijsdijk et al., 2013** | Incident cancers identified through linkage with the Netherlands Cancer Registry and classified according to ICD-10. The oral cancer outcome comprised **lip, oral cavity, and pharynx (ICD-10 C00–C14)**. |
| **Choe et al., 2021** | Incident cancers identified through the Korean National Health Insurance Service database using ICD-10 codes. The oral cancer outcome comprised **lip, oral cavity, and pharynx (ICD-10 C00–C14)**. |
| **Choi et al., 2023** | Incident oral cavity cancer identified through the Korean National Health Insurance Service database using standardized ICD-10 coding for oral cavity cancer. |
| **Lin et al., 2025** | Incident cancers identified through linkage with national cancer registries and classified according to ICD-10. The oral cancer outcome comprised **lip, oral cavity, and pharynx (ICD-10 C00–C14)**. |
